# The Relevance of Otorhinolaryngology in Social and Economical Vulnerability Scenarios and its Role in Medical Training: Experience of FMUSP's Bandeira Científica in 2025

**DOI:** 10.1055/s-0046-1829632

**Published:** 2026-11-04

**Authors:** Lucas A. Chinelatto, Mateus Simões, Ruan S. M. Siqueira, Beatriz Tiemi Mazzer Okuyama, Ricardo Ferreira Bento

**Affiliations:** 1 Department of Otorhinolaryngology, Faculdade de Medicina, Universidade de São Paulo (USP), São Paulo, SPBrazil

## Introduction

Bandeira Científica is an educational extension project of the Faculdade de Medicina da Universidade de São Paulo (FMUSP). It has the purpose of promoting humanitarian expeditions throughout Brazil. In 2025, Marajó Island was elected as the expedition destination, with activities in the cities of Salvaterra and Soure, in the state of Pará. In total, 57 students, ranging from the 2nd to the 5th year of medical school, and 37 supervising physicians, including 1 3rd-year otorhinolaryngology resident from USP's Hospital das Clínicas took part in this expedition.

Medical appointments in Salvaterra took place in a children's daycare center, which was rearranged to provide consultations ( Fig. 1 ). In Soure, consultations initially began in two Primary Care Units, but were later relocated to a kindergarten school, with similar infrastructure adaptations done in Salvaterra. Providing care in adapted schools allowed the expedition to have a greater number of consultation offices—as schools are bigger than most health care units—while also decreasing the impact on the local health care appointments. Nonetheless, the educational impact for the building's students is minimal, as the expeditions occur during summer vacations.

**Fig. 1:**
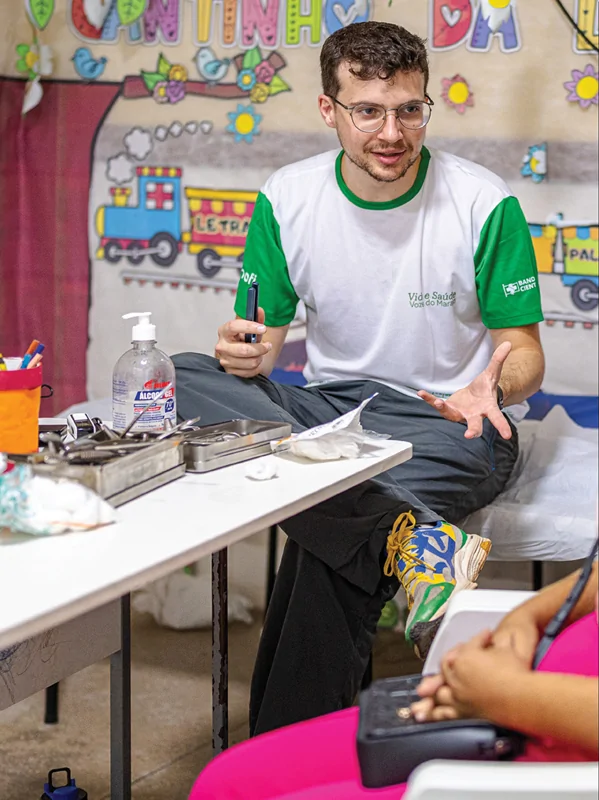
Salvaterra's appointment station. In picture, one of the authors is explaining the plan of care for the patient (not shown in figure).

The appointments consisted of both scheduled cases—arranged either by the Municipal Health Department or by the Bandeira Científica itself—and open slots for spontaneous demands. Consultation dynamics consisted of students conducting triage and consultation in temporary offices adapted from classrooms. Then, the cases were presented to the supervising physician, who evaluated the medical history, explained the diagnostic hypotheses and fundamental concepts for care of the ongoing case. If necessary, the patient was reexamined by the attending physician and student.

Within otorhinolaryngology, where there was only one supervisor, care was provided on alternating days between the two cities, aiming to guarantee access to the specialty in both locations.

## Health Care Impact

During 7 days of service, 110 specialized ear, nose and throat (ENT) consultations were performed, with an average of 16 patients per day. Complaints were mostly otological ( Fig. 2 ). The demographic analysis of the population in the study showed a diverse profile, with a mean age of 32 years, comprising 26.3% children and 10.91% elderly (> 65-years-old).

**Fig. 2:**
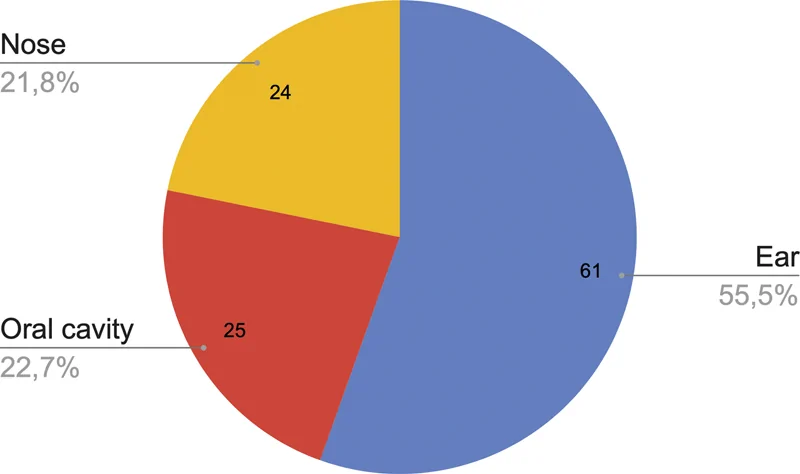
Percentage of all cases by area of complaint.

Marajó Island lacks access to specialized healthcare: although 78.2% ENT patients attended regularly a primary care unit, 71.8% had never been evaluated by an otorhinolaryngologist ( Fig. 3 ). This difficulty in access is partially due to the transport logistics required to see a specialist in Belém. To attend an otorhinolaryngology consultation outside of the expedition, patients face a grueling journey: approximately 2 hours and 30 minutes of travel by boat, with total costs (transportation, food, and lodging) ranging between 30 USD and 40 USD—prohibitive values for a large proportion of the local population. 1

**Fig. 3:**
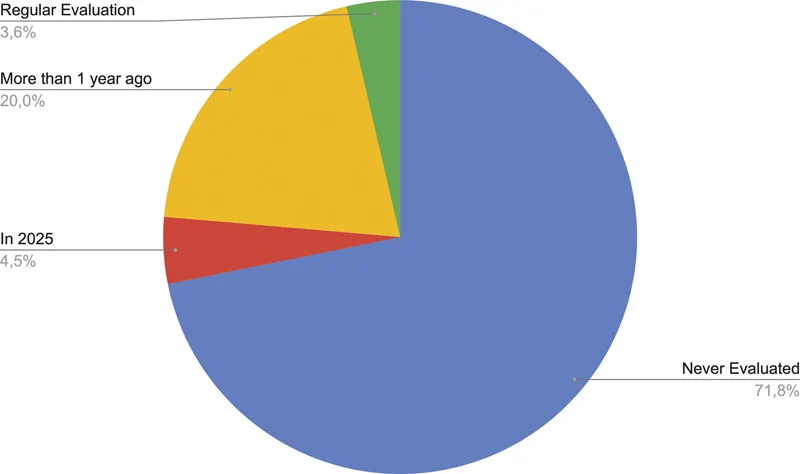
Existence of a previous follow-up with a specialist doctor (otorhinolaryngologist).

The physical and financial barrier is reflected in the chronification of health conditions: 74.5% of the complaints were chronic, with a significant impact on quality of life and a mean score of 6 on the visual analog scale (VAS). Among the main diagnoses made, hearing loss, reflux, rhinosinusitis, and dysphonia stood out ( Table 1 ). The finding of hearing loss as a leading diagnosis in the population of the study reaffirms the conclusions of the 2015 Global Burden of Disease, in which hearing loss was found to be the fourth most common chronic clinical condition globally, affecting up to 1.2 billion people. 2 From the perspective of acute complaints, most of the cases left unsolved by local doctors involved otological complaints ( Table 2 ).

**Table 1:** Main chronic diagnoses

Chronic diagnoses		N
Hearing loss	Bilateral	6
	Presbycusis	8
	Congenital	5
	Traumatic	1
Rhinitis		11
Reflux		11
Rhinosinusitis		7
Dysphonia		7
COM		7
Dizziness		6
Tonsils and adenoids hypertrophy		6
Tubal insufficiency		4
OME		2
Septal deviation		2
Others		5

**Abbreviations:** OME, otitis media with effusion; COM, chronic otitis media.

**Table 2:** Main acute diagnoses

Acute diagnoses	Nº
Cerumen	8
Acute otitis externa	3
Eczematous otitis externa	3
AOM	2
Superior airway infection	2

**Abbreviation:** AOM, acute otitis media.

Despite the limitations in infrastructure and access to complementary exams (such as audiometry, nasofibroscopy, and computed tomography), clinical effectiveness reached 60%, demonstrating that low-complexity interventions, such as ear lavage and assertive therapeutic guidance, have a transformative impact on the local reality.

## Educational Impact

Beyond the healthcare benefits and prospects that otorhinolaryngology represents within the Bandeira Científica project, it is essential to recognize its significant impact in students' training. 3 Regardless of their individual progress in the medical course, the contact with this specialty was able to induce reflections, spark curiosity in consolidating knowledge, and increase students' interest in this field.

For second-year undergraduates, it was the first contact with specific cases from this medical area. Therefore, it provided an opportunity to understand and explore them while considering patients history, seeking to understand how their disease could impact their daily lives. After that, they were able to expand their understanding even further through clinical discussions with a teacher (third year ENT resident). In these moments, it was possible to unite the past lessons of anatomy and physiology with the diverse clinical manifestations that they encountered. In this manner, students were able to witness the importance of the basic medical and scientific knowledge they were learning during that year.

For third-year students, it was a chance to review and put into practice the theory recently acquired in the specialty's undergraduate discipline. Thus, for the first time, they were able to exercise the clinical reasoning related to the diverse complaints introduced by the patients. With the resident's support, they were able to perform the different procedures and steps of otorhinolaryngological semiology, in addition to reinforcing differential diagnoses and increasing their capacity to reach the correct diagnosis for each case.

Regarding students in a more advanced stage of undergraduate training, such as the fifth year, this contact with the specialty had the capacity to reinforce concepts acquired throughout the theoretical and practical learning of medical school. Through the systematic repetition of the specific semiology, especially otoscopy, it was possible to expand technical confidence and the interpretation of findings. Regarding clinical discussion, it allowed for a syndromic approach to the health conditions presented by the patient, which stimulates diagnostic reasoning and the interpretation of different complaints and clinical profiles, such as otitis, tinnitus, as well as conductive and sensorineural hearing loss.

## Conclusion

The presence of otorhinolaryngology in humanitarian projects, in addition to bringing specialists to a population with cumulative demand, promotes the diffusion of the specialty and the consolidation of knowledge useful to any generalist when inserted within the context of academic-educational projects. The specialty brings the advantage of allowing practical contact with a specific propedeutics, and presents territory for diagnostic and teaching expansion within humanitarian medicine using rigid scopes, nasofibroscopies, and even simplified audiometric exams.

The findings of this project demonstrate unquestionable benefits of the specialty's presence in humanitarian health expeditions: specialized care reduces suffering of an unassisted population, and medical education is powered by the presence of a specialty that is briefly presented in the undergraduate core curriculum.
